# Early results of total hip arthroplasty using dual-mobility cup in patients with osteonecrosis of the femoral head

**DOI:** 10.1051/sicotj/2018001

**Published:** 2018-02-21

**Authors:** Chahine Assi, Nadim Kheir, Camille Samaha, Pascal Kouyoumjian, Kaissar Yammine

**Affiliations:** 1 Department of Orthopedic Surgery, Lebanese American University-Rizk Hospital, Beirut Lebanon; 2 Department of Orthopedic Surgery, Middle East Institute of Health, Bsalim Lebanon; 3 Department of orthopedic Surgery, Centre Hospitalier Universitaire de Nîmes, Nîmes France; 4 Center for Evidence-based Anatomy, Sports & Orthopedic Research, Jdeideh Lebanon

**Keywords:** total hip arthroplasty, dual mobility cup, revision surgery, dislocation, survivorship

## Abstract

*Introduction*: Osteonecrosis of the femoral head (ONFH) remains a therapeutic challenge for patients undergoing total hip arthroplasty (THA). The majority of these patients are young with high functional demand, and show an increased risk of dislocation following surgery than patients with osteoarthritis. The use of double mobility cup (DMC) has been linked with lower rates of complications when compared to conservative cups; however, the literature is scarce over DMC results in patients with ONFH. The aim of the study is to report the early outcomes of patients with ONFH treated with THA-DMC. *Materials*: A retrospective analysis of patients suffering from ONFH who underwent THA using DMC (THA-DMC) from 2006 to 2015 were evaluated for functional status and risk of post-operative complications. Thirty THA-DMC in 26 patients with a mean follow-up of 51 months were evaluated clinically (modified Hip Harris Score) and radiologically. *Results*: The mean age of the included patients was 54.9 years. At final follow-up, the mean modified Hip Harris score was 98.7 ± 2.7 and no dislocation episodes or revision surgeries were recorded. The radiological assessment revealed no signs of migration/tilting, radiolucent lines, periprosthetic osteolysis or heterotopic ossification over the DMC component and the femoral stem. The survival rate over 51 months of follow-up was 100%. *Discussion*: The use of the new generation of dual mobility cup in patients with ONFH showed excellent functional early results with no major complications such as dislocation.

## Introduction

Osteonecrosis of the femoral head (ONFH) is defined when cellular death of bone occurs due to disruption of the blood supply that leads to the collapse of the architectural boney structure [1]. This will eventually lead to articular cartilage damage and osteoarthritis of the joint [1]. The four main etiologies of ONFH are idiopathic, dyslipidemia, chronic alcoholism, and corticosteroid treatment [2]. During the early stages of the disease, conservative treatments have been advocated [3]. As articular damage progresses and loss of articular congruency ensues, THA is indicated in these patients.

ONFH remains a therapeutic challenge in this patient population. The fact that most of these patients are young and have high functional demand, the risk of dislocation following THA in ONFH compared to THA for primary osteoarthritis (OA) is higher [4]. It is known that these patients have less favorable outcomes [5] due to higher risk of post-operative complications, functional status, or revision rate [6]. It has been also reported that dislocation is the most common cause of THA revision surgery during the first five years and those with ONFH had the highest risk for revision surgery [7]. Thus, it is critical to choose the appropriate THA implant for this population, which ensures stability, mobility, solidity, and longevity [8].

The dual mobility cup (DMC) concept introduced by Gilles Bousquet in 1974 is known for its low dislocation rates [9,10] with high range of motion [11,12] which fits the specifications needed in patients suffering ONFH. Classically, the DMCs were indicated for patient with short life span, elderly patients > 70 years old, or in cases of muscular or neurological deficiencies [13] due to the complications pertained in the 1^st^ generation dual mobility cups. These complications included premature wear of the polyethylene (intra-prosthetic dislocation), insufficient means of bony fixation and iliopsoas tendon impingement [14,15]. However, with recent advancements in the 2^nd^ and 3^rd^ generations DMCs' design they have proven to yield lower mechanical complications when compared to conventional cups (CC) [16]. The only study published by Martz et al. showed that young patients suffering from ONFH who underwent THA with DMC showed a survival rate of 100% at 10 years follow-up [8].

The aim of this study is to evaluate the early functional status and dislocation rate of a consecutive series of patients suffering from advanced ONFH that underwent THA with DMC.

## Material and Method

This is a retrospective analysis of prospectively collected data of patients suffering from ONFH who underwent primary THA using DMC between Jan 2006 till June 2016. Prior to the study, the ethical boards from the two institutions where the study has been conducted approved the research project. In all cases, the patient's consent has been obtained. Inclusion criteria were set as follows; (a) primary ONFH, (b) ONFH stage 3 and 4 of Arlet and Ficat [17] and (c) a follow-up of minimum of 2 years. Exclusion criteria were the presence of a neurological motor disease, debilitating disease, and history of hip fracture/ infection. Demographic data, etiology, type of approach, type of stem/acetabular cup, bearing surface and coating, and last follow-up were recorded. The posterolateral approach was used systematically. All THA had a cemented femoral stem and a press-fit DMC of 2^nd^ or 3^rd^ generation.

Primary outcomes were set to be the dislocation rate and functional status. The secondary outcomes were defined as the survivorship, infection rate and radiological results. The clinical and radiological data were collected for evaluation. Patient's who were unable to attend for follow-up appointments were contacted by phone and asked to send new radiograms. The modified Harris Hip Score (mHHS) was used for clinical assessment. Migration/tilting, radiolucent lines, periprosthetic osteolysis and heterotopic ossification were recorded on last radiograms. Data collection and analysis were performed by a blinded evaluator.

## Results

Twenty six patients with primary ONFH were treated with THA using DMC during the study period. The sample included 30 DMC comprising 12 males (14 DMC) and 14 females (16 DMC). The mean age of the patients was 54.9 years (SD ± 17.5), ranging between 25 and 90 years. An equal number of 15 DMC was performed on each side. The demographic characteristics of the sample are listed in Table 1.

**Table 1:** Characteristics of the sample

Number of patients = 26	Number of THA = 30
Age (years; mean ± SD)	54.9 ± 17.5
Sex Ratio (female/male)	14/12
Etiology (per hip)	
1) Steroids	8 (26.7%)
2) Idiopathic	10 (33.3%)
3) Smoker	1 (3.3%)
4) SCD	1 (3.3%)
5) CKD	1 (3.3%)
6) HIV Meds	2 (6.7%)
7) Steroid/alcohol	4 (13.3%)
8) Steroid/smoking	1 (3.3%)
9) Smoking/alcohol	2 (6.7%)
Mean mHHS	98.7 (SD ± 2.7)

The used DMC implants were as follows; 27 Avantage, 2 Quattro and 1 HNG. All heads were metallic. A 22.2 mm femoral head was used in 4 subjects (5 hips) and a 28 mm for the remaining patients.

One patient had a traumatic femoral peri-prosthetic fracture after 1.5 years treated with internal fixation (final mHHS = 96). There was no dislocation event throughout the follow-up period. The survival rate of this DMC series was found to be 100% in a range of 2 to 10 years. No post-operative infection has been recorded.

**Figure 1:**
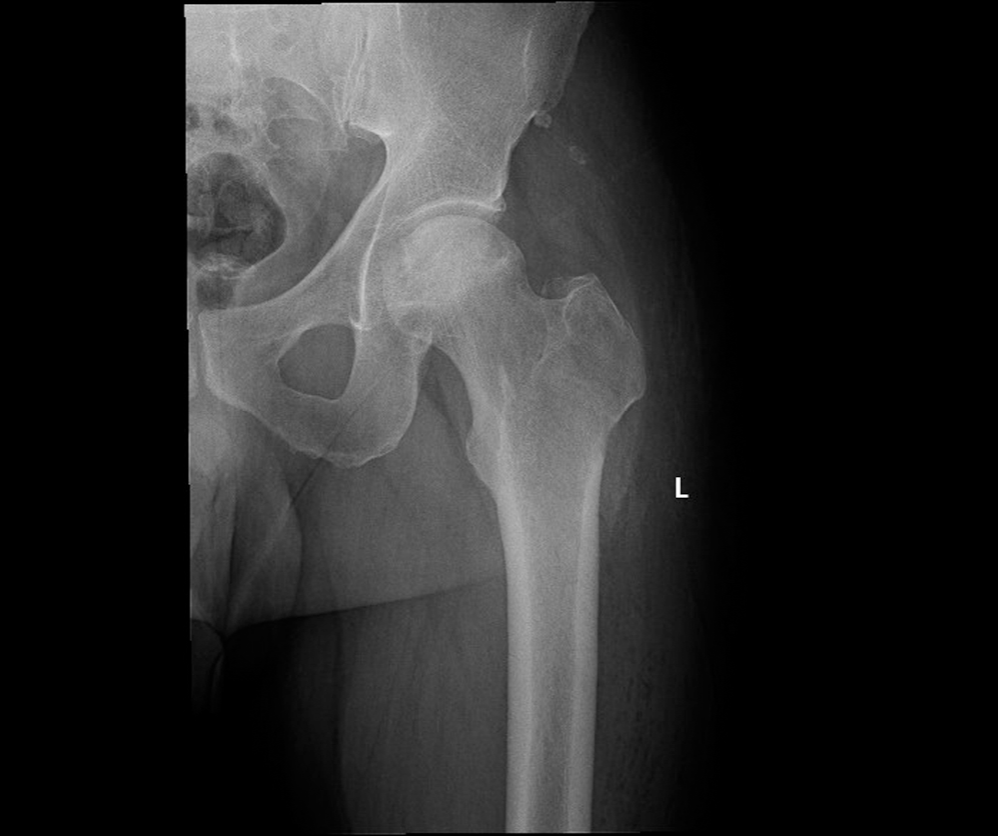
Pre-operative X-ray.

**Figure 2:**
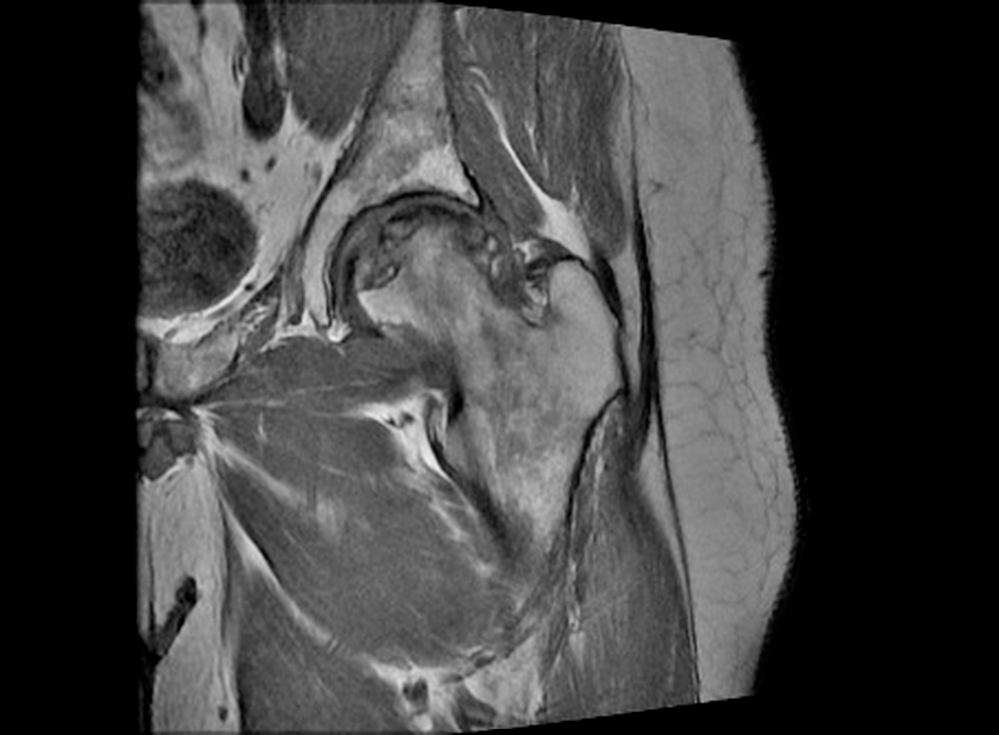
Pre-operative MRI.

**Figure 3:**
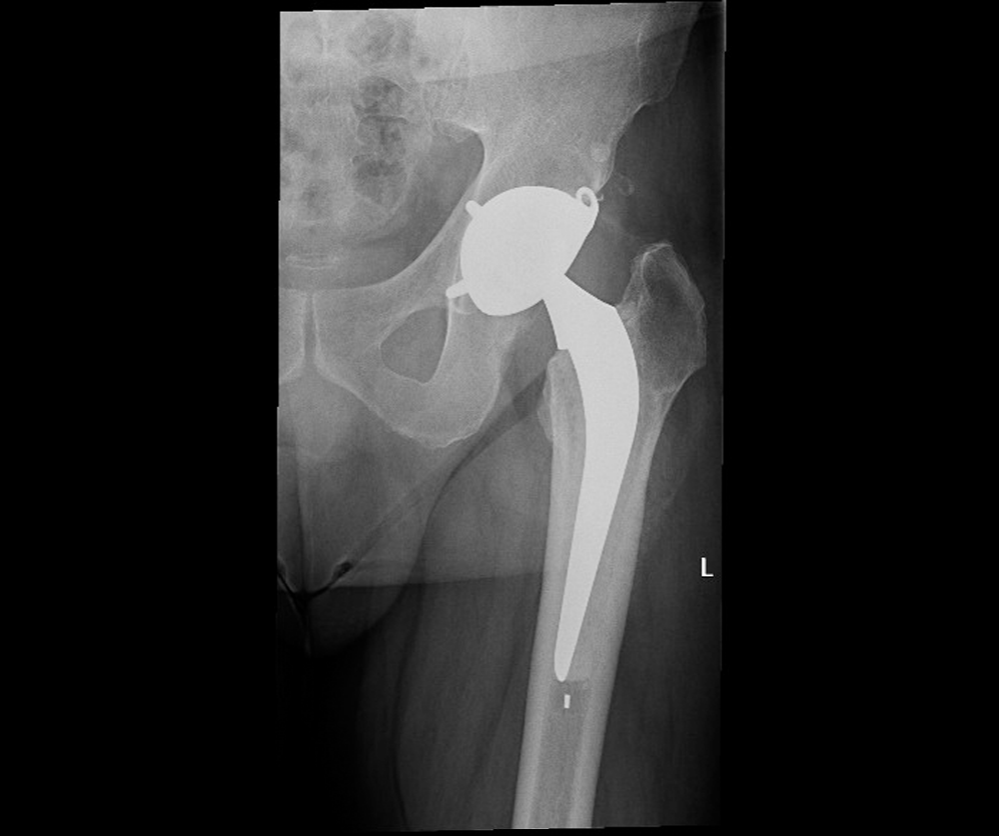
Post-operative X-ray.

Functional and radiological data were collected with a mean follow-up of 51 months (SD ± 28.5). The mean mHHS was 98.7 ± 2.7 (out of a maximal score of 100) ranging from 89 to 100 (upper quartile = 100, lower quartile = 100). Twenty (76.7%) had a mHHS of 100 and the remaining seven (23.3%) patients had a mHHs between 89 and 100. When both groups were compared, no statistical difference was found in relation to femoral head size, age or duration of follow-up. All 26 patients resumed their daily normal activities. At the last follow-up, the radiological assessment revealed no signs of migration/tilting, radiolucent lines, periprosthetic osteolysis or heterotopic ossification over the DMC component and the femoral stem (Figure 1–3).

Lifestyle and religious habits play an integral part in the daily lives in the Middle Eastern region. Prayer rituals performed require extreme degrees of flexion and rotation around the hip (kneeling and trunk bending). In our sample, 38% of the patients were considered religious and were experiencing pain during their prayer pre-operatively. However, at three months of follow-up, these practicing patients had a mean mHHS of 95 ± 2.5.

## Discussion

To our knowledge this is the second research paper over the use of DMC in treating ONFH. Additionally, this is the only case series reporting the results of DMC for ONFH in a Middle Eastern population.

This study demonstrated no episode of dislocation and an excellent survivorship of 100% with a mean follow-up of 51 months. This is in line with the study of Martz et al. (2017) who reported same survival rate at a mean follow-up of 109 months [8]. Similarly, no other major post-operative complications occurred in our series as well; there were no signs of implant loosening or peri-prosthetic osteolysis [8]. While no post-operative infection or ossification were found in our series, Martz et al. [8] reported a single acute post-operative peri-prosthetic infection and a single case of heterotopic calcification of the greater trochanter.

All patients showed an excellent functional score at the last follow-up. The functional status of the patient who experienced a post-traumatic peri-prosthetic femoral fracture in our series had an excellent final functional score as well.

Survivorship of THA with CC (THA-CC) has evolved from rates of 93% in all patient categories [18] to 96.8% in patients less than 50 years old [19]. On the other hand, THA historically showed high failure rates when performed in patients with advanced ONFH reaching up to 30% at ten years of follow-up [20,21]. With the advancement of THA-CC, the survival rate has reached up to 90% at 15 years follow-up for such patients [22,23]. Regardless of THA design improvement, patients with ONFH showed higher revision rates than patients with osteoarthritis and younger patients were at an increased risk than elderly patients [6,7].

Dislocation is known to be the most common cause of revision surgery in the first 5 years, and that regardless the etiology or patient age. However, when compared to THA-CC, a significant difference was demonstrated in favor of DMC in relation to dislocation rates [24]. Using the Lithuanian registry, Tarasevicius et al. compared the survivorship and risk of dislocation in THA-DMC versus THA-CC [25], all etiologies combined. At 5 years of follow-up, the survivorship was 94.8% and 96.1% in the THA-CC and THA-DMC, respectively [25]. The risk of dislocation in the THA-DMC group was 0.7% when compared to 2.4% in the THA-CC [25]. However, in the THA-DMC group the dislocations were intra-prosthetic in nature which was one of complications of the earlier THA-DMC [25].

With regard to ONFH, Hailer et al. [4] demonstrated a 3.7 times greater risk of dislocation in patients with ONFH than in patients with OA when using THA-CC. Two recent studies [9,10] including a number of patients with ONFH demonstrated significant decreased risk of such complication when using THA-DMC, but no subgroup results were reported. On the other hand, Epinette et al. [13] and Assi et al. [26] showed excellent survivorship with no episode of dislocation in young patients treated with THA-DMC. The only published study on ONFH reported excellent results when treated with THA-DMC [8] and our findings are in line with those found by Martz et al. [8]; no episode of dislocation with a 100% survivorship.

When comparing THA-DMC with THA-CC, no statistical difference was found between both groups regarding PE wear, revisions for osteolysis/wear and loosening [24]. Our study showed none of those complications during 45 months of follow-up. In relation to the cemented femoral stem outcome, no radiological signs of loosening at last follow-up were noticed. That was not the case in Martz et al. study [8] in which 11/16 patients (68.75%) had signs of premature loosening in cemented femoral stems at follow-up.

This study is in line with previous studies reporting excellent results of DMC in relation to stability and survivorship in high risk populations such as in hemiplegic patients [27], patients with displaced femoral neck fractures [28] and those who take their hips into hyperflexed position while sitting and praying [26,29].

The limitations of the study are mainly its small sample size and its retrospective design. However, while all 26 patients (30 THA) have been included in our analyses, 11 out of 31 patients (25 out of 40 THA) were lost to follow-up as reported in the study of Martz et al. [8]. In this and to this date, our series is considered the largest in using DMC in patients with ONFH.

In conclusion, the use of the new generation of dual mobility cup in patients with ONFH showed excellent functional and radiological early results with no episode of dislocation or revision. Despite the fact that such patient population is known to have less favorable outcomes, this study showed comparable results with patients having primary osteoarthritis treated with THA-DMC. Thus, the use of DMC is a promising effective and safe solution in high-risk patients such as those with ONFH.

## Financial support

No financial support was received.

## Conflict of interest

The authors declare that the research for and communication of this independent body of work does not constitute any financial or other conflict of interest.
