# Correction notice to: Early results of total hip arthroplasty using dual-mobility cup in patients with osteonecrosis of the femoral head: SICOT-J 2018, **4**, 4, https://doi.org/10.1051/sicotj/2018001

**DOI:** 10.1051/sicotj/2018039

**Published:** 2018-09-03

**Authors:** Chahine Assi, Nadim Kheir, Camille Samaha, Pascal Kouyoumdjian, Kaissar Yammine

**Affiliations:** 1 Department of Orthopedic Surgery, Lebanese American University-Rizk Hospital, Beirut Lebanon; 2 Department of Orthopedic Surgery, Middle East Institute of Health, Bsalim Lebanon; 3 Department of orthopedic Surgery, Centre Hospitalier Universitaire de Nîmes, Nîmes France; 4 Center for Evidence-based Anatomy, Sports & Orthopedic Research, Jdeideh Lebanon

The name of the third author “Pascal Kouyoumjian” has been corrected to “Pascal Kouyoumdjian”. The publisher apologizes for any inconveniences.

